# The Psychiatric Misdiagnosis of Behavioral Variant Frontotemporal Dementia in a Colombian Sample

**DOI:** 10.3389/fneur.2021.729381

**Published:** 2021-11-15

**Authors:** Lina Zapata-Restrepo, Juan Rivas, Carlos Miranda, Bruce L. Miller, Agustín Ibanez, Isabel E. Allen, Katherine Possin

**Affiliations:** ^1^Global Brain Health Institute, University of California, San Francisco, San Francisco, CA, United States; ^2^Trinity College Dublin, Dublin, Ireland; ^3^Hospital Departamental Psiquiátrico, Universitario del Valle, Cali, Colombia; ^4^Department of Psychiatry, Universidad del Valle, Cali, Colombia; ^5^Department of Psychiatry, Fundación Valle del Lili, Cali, Colombia; ^6^Department of Psychiatry, Universidad ICESI, Cali, Colombia; ^7^The Memory and Aging Center, Department of Neurology, Weill Institute for Neurosciences, University of California, San Francisco, San Francisco, CA, United States; ^8^Cognitive Neuroscience Center (CNC), Universidad de San Andrés, Buenos Aires, Argentina; ^9^National Scientific and Technical Research Council (CONICET), Buenos Aires, Argentina; ^10^Latin American Brain Health Institute (BrainLat), Universidad Adolfo Ibáñez, Santiago de Chile, Chile; ^11^Department of Epidemiology and Biostatistics, University of California, San Francisco, San Francisco, CA, United States

**Keywords:** frontotemporal lobar degeneration, frontotemporal dementia, behavioral variant frontotemporal dementia, BPSD (behavioral and psychological symptoms in dementia), neuropsychiatric symptoms in dementia, dementia caregivers, psychiatric misdiagnosis, frontotemporal dementia (FTD) spectrum

## Abstract

**Objective:** To describe the demographic characteristics, initial psychiatric diagnoses, and the time to reach a diagnosis of probable behavioral variant frontotemporal dementia (bvFTD) in a public psychiatric hospital in Cali, Colombia.

**Methods:** We retrospectively reviewed the medical records of 28 patients who were diagnosed with probable bvFTD based on a multidisciplinary evaluation that included a structural MRI, neuropsychological testing, functional assessment, and neurological exam. Prior to this evaluation, all patients were evaluated by a psychiatrist as part of their initial consultation at the hospital. The initial consultation included the Neuropsychiatric Inventory and diagnoses based on the DSM-V. Demographics, clinical features, and initial psychiatric misdiagnoses were extracted from clinical records and summarized in the full sample and by gender.

**Results:** The study sample had a mean education of 10.0 years (SD = 4.9) and 68.0% were female. In the full sample, 28.6% were initially diagnosed with dementia, and 71.4% with a psychiatric disorder. The psychiatric diagnosis at initial consultation differed by gender. Women were most likely to be diagnosed with depression (26.3%) or bipolar disorder (26.3%), while the men were most likely to be diagnosed with anxiety (33.3%) or a psychotic disorder (22.2%). Psychotic symptoms were common (delusions, 60.7% and hallucinations, 39.3%), and the pattern of neuropsychiatric symptoms did not differ by gender.

**Conclusions:** This is one of few case series of bvFTD in a Colombian population, where bvFTD is a recognizable and prevalent disorder. In this psychiatric hospital, the majority of patients with bvFTD were initially diagnosed with a primary psychiatric condition. There was a gender difference in psychiatric diagnosis, but not in neuropsychiatric symptoms. In this sample, the rate of psychiatric misdiagnosis, as well as the psychotic symptoms, were higher compared to rates described in other countries. These results highlight the need for interventions to improve bvFTD diagnosis in under-represented populations.

## Introduction

The clinical presentation of the behavioral variant of frontotemporal dementia (bvFTD) may include alterations in behavior, mood, or changes in personality, language, and motor symptoms (1). Psychiatric symptoms frequently precede cognitive manifestations and commonly include behavioral disinhibition, apathy, and obsessive-compulsive behaviors (2). Psychotic symptoms are seen in the early stages of the disorder in 10 to 32% of patients, with a higher frequency in patients with familial forms of the disease (3–5) and in younger patients with a family history of mental illness (6). In high income countries, bvFTD patients are often initially misdiagnosed as major depressive disorder, bipolar affective disorder, or schizophrenia (7–10). For example, a retrospective chart review of 69 patients with bvFTD diagnosis at a neurology clinic in San Francisco, CA found that 51% had an initial psychiatric diagnosis, and this misdiagnosis was more common for women than men (7). Despite the predominance of psychiatric symptoms in bvFTD, most case series come from teams led by neurologists. Furthermore, most of the studies of bvFTD have focused on people from high income countries (11), and there have been few studies from Central or South America.

Distinguishing patients with bvFTD from patients with primary psychiatric disorders (PPD) is key, because of the drastically different prognosis, differences in patient treatment, family counseling and caregiver education, and the necessity to accurately identify patients with bvFTD in the early stages for future clinical trials (12). Misdiagnosis can delay an early and appropriate diagnosis, prevents adequate support for caregivers, delays the performance of specific medical examinations, genetic counseling, and adequate patient management. It also carries financial risks for patients and their families (13, 14). The bvFTD-PPD differentiation seems to be more challenging in Latin America, as in comparisons with other region such as US or Europe, the health professionals receive less specific training around this condition (15), have less access to biomarkers (16), and the caregivers can experience more burden as consequences of misdiagnosis (15). The goal of the present study is to describe the clinical presentation characteristic of bvFTD in a Latin American population that has been under-represented in research. In Cali, the population is admixed and diverse, with African, Indigenous, and European origins. We focused on the demographics, the neuropsychiatric symptoms, the initial psychiatric misdiagnoses and the time interval it took to reach the correct diagnosis.

## Methods

### Participants

We retrospectively reviewed the medical records of all the patients (*n* = 28) diagnosed with probable bvFTD (19 women and 9 men) at the Hospital Departamental Psiquiátrico Universitario del Valle (HDPUV) in Cali, Colombia in the last 5 years. 36.7% of the patients were referrals from other institutions in the same region. 64.3% of the patients consulted directly to the HDPUV. The diagnosis was made using the diagnostic criteria for probable FTD of the international Behavioral Variant FTD Criteria Consortium (2) and based on results from a neurological exam, functional assessment, neuropsychological testing, and brain imaging; all participants had structural MRI, 14 had a fluorodeoxyglucose (FDG)-positron emission tomography (FDG-PET) and 11 had a single-photon emission computerized tomography (SPECT). Neuroradiologists interpreted the neuroimaging, and they reported frontal and anterior temporal atrophy in the cerebral MRI, frontal and anterior temporal hypometabolism in the FDG-PET, and frontal and anterior temporal hypoperfusion in the SPECT. All the patients presented at least with 3 of the behavioral/cognitive symptoms of the consortium criteria. The results and the diagnosis were discussed in a consensus group made of one neuropsychiatrist, one psychiatrist and one neuropsychologist. The research was approved by the ethics committee at the HDPUV, which is a secondary care public psychiatric hospital in the southwest region of the country. At this hospital, the specialists are psychiatrists, and it embraces a diverse population of patients with low education and socioeconomic status.

### Assessment of Demographics, Clinical Features, and Neuropsychiatric History

For each patient we reviewed all medical records from the psychiatric hospital, which included demographics (age, gender, education, marital status), caregiver information (gender and relationship), family history (family report of dementia in a first or second degree relative to the person), age of symptom onset, and age of bvFTD diagnosis. From the initial consultation by the psychiatrist, we reviewed the DSM-V diagnosis and standardized review of neuropsychiatric symptoms (Neuropsychiatric inventory; NPI) (17). The time intervals examined were the time elapsed since the onset of symptoms and the first time seen by a psychiatrist at the hospital, the time between the first psychiatric diagnosis and bvFTD diagnosis and the time interval between the onset of symptoms and the bvFTD diagnosis.

### Data Analysis

Variables were summarized overall and by gender, and compared using independent groups Student's *t*-test for continuous variables and chi-squared or Fisher's exact test for categorical variables. Results are reported as means and standard deviations for continuous variables and frequencies and percentages for categorical variables. Stata 16.1 and SPSS 27.0 were used for the statistical analysis.

## Results

The sample consisted of 19 women and 9 men, who were similar in terms of age and education. The most frequent caregivers in the case of women were their children (38.8% daughters, 10.5% sons), followed by their siblings (26.3% sisters, 0.0% brothers) and their husbands (26.3%). In the case of men, the group of caregivers was generally represented by their wives (66.7%), followed by their children (22.2% daughters, 0.0% sons), and their mothers (11.9%). Most of the patients evaluated (89.3%), did not report a family history of dementia, and there were no differences based upon gender (Table 1).

**Table 1:** Demographic, caregiver and family history data.

**Variant**		**Female (*N =* 19)**	**Male (*N =* 9)**	**Total (*N =* 28)**	***P* (Student's t)**	***P* (Chi-squared test)**
Age Mean (SD)		63.5 (8.0)	62.1 (11.7)	63.0 (9.1)	0.719	-
Education Mean (SD)		9.5 (5.2)	11.0 (4.9)	10.0 (5.1)	0.467	-
Marital status (%)	Married	47.4	77.8	57.1	-	0.172
	Not married	52.6	22.2	42.9	-	-
Caregiver role (%)	Mother	0.0	22.2	7.1	-	NS
	Sister	26.3	0.0	17.9	-	NS
	Spouse	26.3	66.7	39.3	-	NS
	Child	47.4	11.1	35.7	-	NS
Family history of dementia	Negative	89.5	88.9	89.4	-	NS
	Positive	10.5	11.1	10.7	-	NS

*p-values were calculated using the Student's t-test and the chi-squared test (X^2^). Alpha level set at 0.05*.

During the first visit to the hospital, 71.4% of the patients (73.7% of women and 66.7% of men), were misdiagnosed with a psychiatric disease, and only 28.6% were correctly diagnosed with dementia. In women, the most frequent psychiatric diagnoses were depression (26.3%), and bipolar disorder (26.3%), while in men it was anxiety disorders (33.3%) and psychotic disorders (22.2%). Women were significantly more likely to be misdiagnosed with a bipolar disorder, and men were significantly more likely to be misdiagnosed with an anxiety disorder (Table 2).

**Table 2:** Initial psychiatric diagnostics.

	**Female**	**Male**	**Total**	***p***
	**(*N =* 19)**	**(*N =* 9)**	**(*N =* 28)**	
Dementia (%)	26.3	33.3	28.6	NS
Anxiety disorders (%)	5.3	33.3	14.5	0.045
Depressive disorders (%)	26.3	11.1	21.4	NS
Bipolar Affective disorders (%)	26.3	0.0	17.9	0.032
Psychotic disorders (%)	15.8	22.2	17.9	NS

*Percentage of the initial diagnosis during their first psychiatric visit, separated by gender. p-values were calculated using the chi-squared test (X^2^). Alpha level set at 0.05*.

On average, the age of onset of symptoms, based on medical chart review, was 54.9 years. The age of the first consult averaged 57.0 and the age of the FTD diagnosis was 59.3 years (Table 3). The time elapsed between the onset of symptoms and the first consultation at the hospital averaged 2.1 years (2.6 for women, 1.1 for men). The time between the first psychiatric consultation and the diagnosis of probable FTD was 2.3 years (2.2 for women, 2.3 for men). On average, the time elapsed between the onset of symptoms and the diagnosis of probable FTD was 4.4 years, (4.8 for women, 3.4 for men; Table 3).

**Table 3:** Time lapses between onset of symptoms and diagnosis [Mean (SD)].

	**Female** **(*N =* 19)**	**Male** **(*N =* 9)**	**Total** **(*N =* 28)**
Age onset of symptoms (Time in years)	54.5 (7.9)	55.7(10.8)	54.9 (8.7)
Age first consult (Time in years)	57.1 (8.3)	56.8(10.5)	57.00 (8.8)
Age diagnosis of probable bvFTD (Time in years)	59.3 (8.5)	59.1(10.7)	59.3 (9.0)
Onset Symptoms—1st consult (Time in years)	2.6 (2.5)	1.1(0.8)	2.1 (2.2)
1st consult—bvFTD diagnosis (Time in years)	2.2 (1.2)	2.3(2.1)	2.3 (1.5)
Total time (onset symptoms—bvFTD diagnosis) (Time in years)	4.8 (2.7)	3.4(2.3)	4.4 (2.6)

The diagnosis was made based on the criteria of the international consortium (2) with a total average of 4.1 (0.7) criteria in the case of women and 4.2 (0.7) in the case of men. In all cases, the patients presented with apathy (Table 4). All women also presented socially inappropriate behaviors and executive dysfunction, which was reported at the neuropsychological assessment. Regarding men, the most frequent criteria, after apathy, were socially inappropriate behaviors, alteration in diet and executive dysfunction.

**Table 4:** Criteria for possible bvFTD.

**bvFTD international criteria**	**Female %**	**Male %**	**Total %**	***p***
	**(*N =* 19)**	**(*N =* 9)**	**(*N =* 28)**	
A.1. Socially inappropriate behavior	100	88.9	96.4	NS
A.2. Loss of manners or decorum	57.9	77.8	64.3	NS
A.3. Impulsive, rash or careless actions	42.1	33.3	39.3	NS
B.1. Apathy	100.0	100.0	100.0	NS
B.2. Inertia	84.2	77.8	82.1	NS
C.1. Diminished response to other people's needs and feelings	89.5	77.8	85.7	NS
C.2. Diminished social interest, interrelatedness or personal warmth	89.5	77.8	85.7	NS
D.1. Simple repetitive movements	15.8	22.2	17.9	NS
D.2. Complex, compulsive or ritualistic behaviors	15.8	11.1	14.2	NS
D.3. Stereotypy of speech	0.0	11.1	3.6	NS
E.1. Altered food preferences	73.7	88.9	78.6	NS
E.2. Binge eating, increased consumption of alcohol or cigarettes	15.8	33.3	21.4	NS
E.3. Oral exploration or consumption of inedible objects	10.5	22.2	14.3	NS
F.1. Deficits in executive tasks	100.0	88.9	96.4	NS
F.2. Relative sparing of episodic memory	26.3	0.0	17.9	NS
F.3. Relative sparing of visuospatial skills	73.7	55.6	67.9	NS

*p-values were calculated through the chi-squared test (X^2^). Alpha level set at 0.05*.

**Figure 1:**
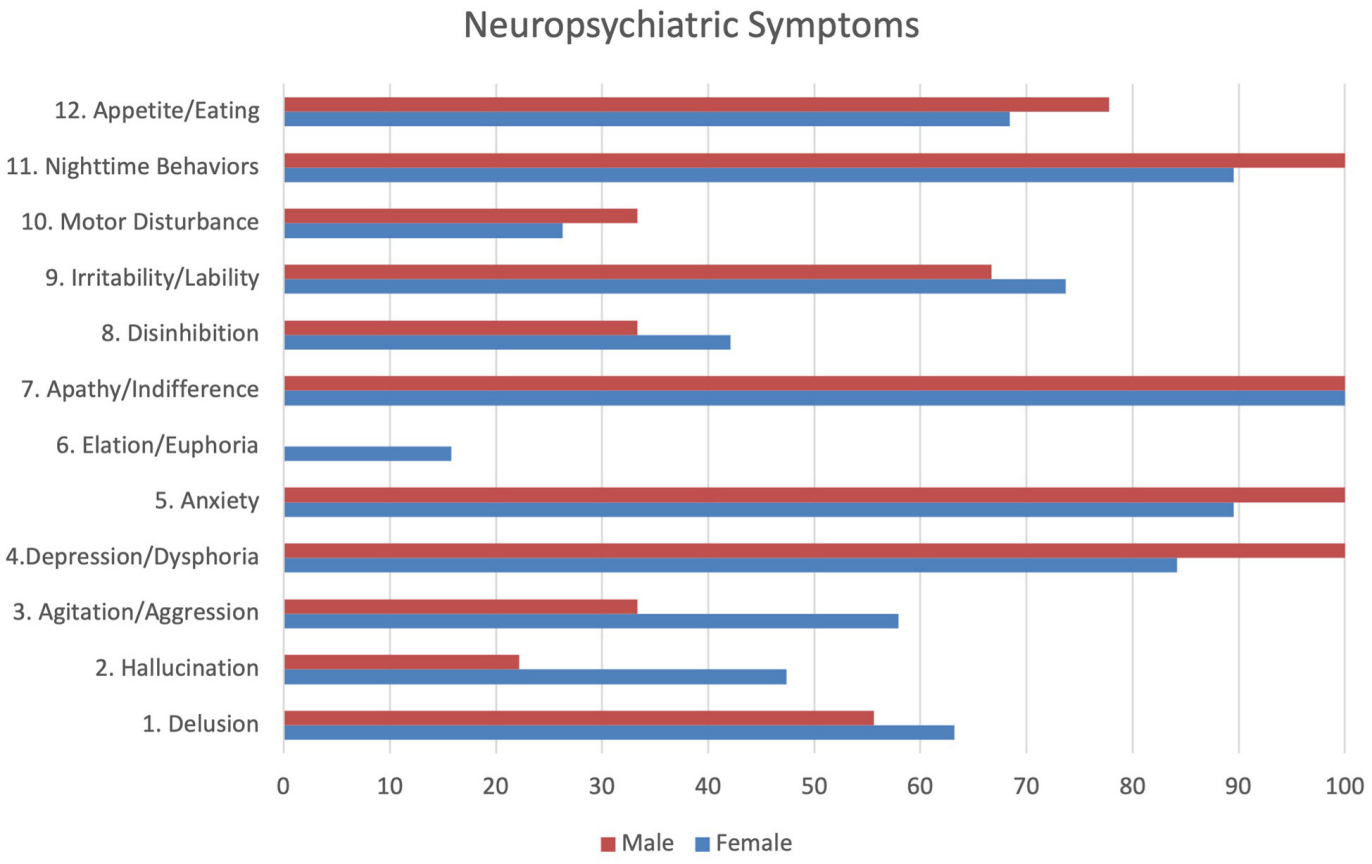
Neuropsychiatric symptoms.

Neuropsychiatric evaluation, using NPI, showed that all patients presented with apathy/indifference. In the case of women, the most frequent symptoms were anxiety, sleep disturbances, and depression/dysphoria. In men, the most common symptoms were depression/dysphoria, anxiety, and sleep disturbances (Figure 1).

## Discussion

We reviewed the medical records of 28 patients who were diagnosed with bvFTD at a public psychiatric hospital in Cali, Colombia and who had undergone a systematic review of neuropsychiatric symptoms at initial presentation to the hospital. We found that 71.4% of the patients were initially misdiagnosed with a psychiatric disorder. Despite similar demographics, bvFTD diagnostic criteria, and neuropsychiatric symptoms between male and female patients, they exhibited different patterns of misdiagnosis. Women were significantly more likely to be misdiagnosed with bipolar affective disorder, and men with an anxiety disorder. Depression was also a common misdiagnosis for women, and psychotic disorder for men.

We also described the demographics and clinical history of our sample. The sample had an average age of 63 years at the time of diagnosis, and an average time from first symptom to diagnosis of 4.3 years, which is consistent with previous studies from North America and Europe (18, 19). We found a gender difference compared to previous reports with more than twice as many women with bvFTD compared to men (3, 20). It is unknown whether this difference represents something unique about the prevalence of bvFTD in this region, or if it is the result of other factors, such as a referral bias. The average educational level of the sample (10 years) was typical for Colombia (21), yet much lower than prior studies of bvFTD cohorts from the United States and Europe (7, 22).

We found high rates of delusions (60.7%) and hallucinations (39.2%), which may be due to our setting at a psychiatric hospital. Psychotic symptoms are more frequent in patients with genetic mutations (38%), than in those without (4%), but psychosis was higher in this study than any genetic cohort previously described (3). The C9orf72 mutation has been identified as the most frequent genetic cause of FTD. The most common psychiatric presentation is psychosis (21–56%), with delusions, and/or hallucinations (23). Hallucinations were present in 12% of patients with FTD in a clinicopathological correlation study, related to TDP-43 pathology including, but not exclusively, C9orf72 (24). Paradoxically, only 10.7% of this sample reported a family history of dementia. A more systematic evaluation of family history and ideally genetic assessment will be required to better establish the potential origin of these psychiatric symptoms. Preliminary results from the Multi-Partner Consortium to Expand Dementia Research in Latin America ReDLat, (25, 26) found several genetic causative mutations in patients coming from Colombia who had no apparent family antecedents of dementia.

The presence of psychiatric symptoms and their possible overlap in patients with bvFTD make differential diagnosis difficult (27). While psychiatric misdiagnosis of bvFTD is common in high-income countries (2, 5), we found an even higher rate. This is likely due, at least in part, to the location of our clinic at a psychiatric hospital, but could be explained by the greater challenges in diagnosis in our population due lower awareness about dementia among the public (16), insufficient professional training (28), limited access to biomarkers (16) or perhaps more frequent psychiatric presentations in this under-represented sample.

In our sample, the majority of caregivers were women. In the case of women patients, the majority of caregivers were daughters while in the case of men, their caregivers were more likely to be their wives. Studies of FTD caregivers' demographics are not extensive in Latin America (15). However, the impact of bvFTD caregiver burden and financial strains in the region seem to be higher than other dementias (29–31), and it appears, as with other dementias, that this burden falls disproportionately on women (32–35). This is the first study we are aware of to report on bvFTD caregiver demographics in Colombia.

Our results underscore the importance of considering a bvFTD etiology for patients with late-onset psychiatric symptoms, with urgent referral to a dementia specialist (when feasible) to reduce delays in diagnosis and care. Based on our study, several red flags can be identified. Diminished social interest and response to others' feelings, which may be misinterpreted as signs of depression, was very common in our sample. In addition, socially inappropriate behavior and loss of manners, which might be interpreted as mania in some cases, was also common. Red flags may also differ by gender such that signs of bipolar affective disorder in women and anxiety in men may suggest review of bvFTD criteria is indicated. Standardized cognitive testing could be valuable given the high rates of executive function impairment and sparing of visuospatial skills.

## Limitations

This is a retrospective study, and the data depend on the quality of the psychiatrist's assessments and records. In addition, all the specialists who evaluated the patients are psychiatrists, which could imply a diagnostic bias, and so results must be understood in this context. Also, the neuropsychology assessment was performed by different neuropsychologists in the city and the evaluation protocol varied, and so these data are not reported. The size of the sample does not allow the results to be generalized and the distribution by gender is not equitable. Additionally, the patients do not have a definite FTLD pathology, since a confirmatory autopsy is not performed, and genetic testing has not yet been performed in our region.

## Conclusions

We reviewed the medical charts of 28 patients with bvFTD at a public psychiatric hospital in Cali, Colombia. It is the main psychiatric hospital in the southwest of the country, were psychiatrists see an underrepresented population with low socioeconomic status and educational level. At the initial evaluation misdiagnosis of bvFTD was high even though these patients met research criteria for bvFTD. Psychotic symptoms were higher in this sample than in previous studies. Regarding the caregiver, the majority are women and there is a difference in the caregiver-patient relationship according to the gender of the patient. Diagnostic pathways for bvFTD should be incorporated into evaluations for adults with behavioral disturbances including use of a family tree. In the future, the implementation of blood and cerebrospinal fluid biomarkers and genetic testing will help to improve the diagnosis of bvFTD. Autopsy programs are also needed. We want to highlight the importance of educational programs and trainings for health professionals in our community, for a better diagnosis of bvFTD in this part of Colombia.

## Data Availability Statement

The original contributions presented in the study are included in the article/supplementary material, further inquiries can be directed to the corresponding author/s.

## Ethics Statement

The studies involving human participants were reviewed and approved by Hospital Departmental Psiquiátrico Universitario del Valle. Written informed consent for participation was not required for this study in accordance with the national legislation and the institutional requirements.

## Author Contributions

LZ-R, KP, JR, CM, and BM contributed to the study concept and design. LZ-R, JR, and CM contributed to data acquisition. KP and IA contributed to the statistics/verified the analytical method. LZ-R, JR, KP, and BM contributed to writing the first draft. LZ-R, KP, and AI supervised the study, read, and approved the final version. All authors contributed to the article and approved the submitted version.

## Funding

AI was partially supported by grants from CONICET; ANID/FONDECYT Regular (1210195 and 1210176); FONCYT-PICT 2017-1820; ANID/FONDAP/15150012; Takeda CW2680521; Sistema General de Regalías (BPIN2018000100059), Universidad del Valle (CI 5316); Alzheimer's Association GBHI ALZ UK-20-639295; and the MULTI-PARTNER CONSORTIUM TO EXPAND DEMENTIA RESEARCH IN LATIN AMERICA [ReDLat, supported by National Institutes of Health, National Institutes of Aging (R01 AG057234), Alzheimer's Association (SG-20-725707), Rainwater Charitable Foundation—Tau Consortium, and Global Brain Health Institute)]. LZ and JR was partially supported by Sistema General de Regalías (BPIN2018000100059), Universidad del Valle (CI 5316).

## Author Disclaimer

The contents of this publication are solely the responsibility of the authors and do not represent the official views of these institutions.

## Conflict of Interest

The authors declare that the research was conducted in the absence of any commercial or financial relationships that could be construed as a potential conflict of interest.

## Publisher's Note

All claims expressed in this article are solely those of the authors and do not necessarily represent those of their affiliated organizations, or those of the publisher, the editors and the reviewers. Any product that may be evaluated in this article, or claim that may be made by its manufacturer, is not guaranteed or endorsed by the publisher.
